# Report on the 3'rd scientific meeting of the "Verein zur Förderung des Wissenschaftlichen Nachwuchses in der Neurologie" (NEUROWIND e.V.) held in Motzen, Germany, Nov. 4'th - Nov. 6'th, 2011

**DOI:** 10.1186/2040-7378-4-2

**Published:** 2012-02-23

**Authors:** Christoph Kleinschnitz, Sven G Meuth, Tim Magnus, Thomas Korn, Ralf A Linker

**Affiliations:** 1 Department of Neurology, University Clinic of Würzburg, Josef-Schneider-Str. 11, 97080 Würzburg, Germany; 2 Department of Neurology-Inflammatory Disorders of the Nervous System and Neuro-oncology, Westfälische Wilhelms-University Münster, Domagkstr. 13, 48149 Münster, Germany; 3 Department of Neurology, University Clinic Hamburg-Eppendorf, Martinistr. 52, 20246 Hamburg, Germany; 4 Department of Neurology, Klinikum rechts der Isar, Technische Universität München, Ismaninger Str. 22, 81675 München, Germany; 5 Department of Neurology, Friedrich-Alexander-Universität Erlangen, Schwabachanlage 6, 91054 Erlangen, Germany

## Abstract

From November 4^th^- 6^th ^2011, the 3^rd ^NEUROWIND e.V. meeting was held in Motzen, Brandenburg, Germany. Like in the previous years, the meeting provided an excellent platform for scientific exchange and the presentation of innovative projects for young colleagues in the fields of neurovascular research, neuroinflammation and neurodegeneration. As kick-off to the scientific sessions, Reinhard Hohlfeld, Head of the Institute for Clinical Neuroimmunology in Munich, gave an illustrious overview on the many fascinations of neuroimmunologic research. A particular highlight on the second day of the meeting was the award of the 1'st NEUROWIND e.V. prize for young academics in the field of experimental neurology. This award is posted for young colleagues under the age of 35 with a significant achievement in the field of neurovascular research, neuroinflammation or neurodegeneration and comprises an amount of 20.000 Euro, founded by Merck Serono GmbH, Darmstadt. Germany. The first prize was awarded to Ivana Nikic from Martin Kerschensteiner's group in Munich for her brilliant work on a reversible form of axon damage in experimental autoimmune encephalomyelitis and multiple sclerosis, published in *Nature Medicine *in 2011. This first prize award ceremony was a great incentive for the next call for proposals now upcoming in 2012.

## Summary of the scientific contributions to the NEUROWIND e.V. meeting 2011

### Contributions on neuroimmunology and neurodegeneration - interactions between the immune system and the nervous system

In 2011, presentations in the field of neuroimmunology dealt with multiple sclerosis (MS), chronic inflammatory demyelinating polyneuropathy (CIDP), antibody-mediated diseases (stiff-person-syndrome - SPS, Batten disease, neuromyelitis optica - NMO, limbic encephalitis) and Shiga toxin (Stx)-producing *Escherichia coli *(STEC) infection.

Anne Willing from Manuel Friese's lab in Hamburg demonstrated that IL-17 production by CD8+ T cells is mainly restricted to CD161 high cells. In MS patients, CD4 + CD161+ cells are significantly increased in the CSF. In contrast CD161 high CD8+ cells belonging to mucosal associated invariant T cells (MAIT) reacting to bacteria but not viruses are significantly reduced in peripheral blood and CSF in MS.

Christina Heinemann from Thomas Korn's laboratory in Munich investigated the role IL-23 in the priming of encephalitogenic T helper cell responses. Using IL-23R knockout mice, she found that a major T cell intrinsic function of IL-23 is the suppression of IL-10. Thus, an overwhelming IL-10 response rather than the lack of Th17 cells in IL-23R KO mice confers resistance to EAE in these animals.

The importance of microRNAs in neuroinflammation was highlighted by Andreas Junker from Göttingen. 172 from 365 tested microRNAs were detected in inactive MS lesions. Conversely, Andreas showed that microRNA-155 was overexpressed in active MS lesions and microRNA155 deficient mice demonstrated an ameliorated EAE disease course.

Another EAE project was presented by Arndt Manzel from Ralf Linker's group in Erlangen. Arndt investigated the role of the sodium/fluid balance in T cell responses. Together with recent data on the role of the renin angiotensin system in autoimmune inflammation, these results further underscore the notion that key players in renal function may also impact on immune responses.

Catherina Sorbara from Martin Kerschensteiner's group in Munich demonstrated the importance of axonal damage for sustained disability progression in MS patients. Using two-photon microscopy of the lumbar spinal cord, she was able to show an increased axonal density of mitochondria in axons of EAE mice compared to controls. This process was interpreted as a transport deficit that might be mediated by NO and/or H_2_O_2 _production.

Sebastian Fiebiger from Berlin (group of Friedemann Paul and Carmen Infante-Duarte) talked about oxidative damage in neuroinflammation focusing on the role of microglia derived NO that targets mitochondria. The sequence of destructive events includes a reduced ATP output and increased ONOO- leading to cytochrome C release and degradation of cytoskeletal proteins. Within this context the antioxidant idebenone - a synthetic analogue of coenzyme Q10 is currently tested in phase I/II trials in PPMS. In Sebastian's studies, idebenone protected HT22 neuronal cells from glutamate induced cell death but failed to influence the disease course of EAE animals.

CIDP is a chronic inflammatory disease of the peripheral nervous system with autoimmune pathogenesis. So far, the auto-antigen is not defined and potentially relevant protein candidates include PMP22 and P0. Martin Russwurm from Marburg (Björn Tackenberg's group) presented a study of quantitative expansions in antigen-specific T cells via TCRvbeta analysis in 24 patients suffering from CIDP using FACS analysis and CDR 3 spectratyping before and after IVIG treatment. In CIDP patients, increased numbers of cells with specific Vbeta domains before IVIG treatment (in 70% of patients) in CD4+ and CD8+ T cells were reduced under IVIG therapy (in particular in CD4+ cells). CDR3 spectratyping analysis suggested that the overrepresentation of specific Vbeta chains was due to oligoclonal expansion of specific T cell clones. Upon immunophenotyping, Vbeta positive patients displayed an increase in CD95 (FAS), VLA-4 and LFA-1 as well as a reduction of CD28 in CD4CD45RO + cells.

Christian Geis from Würzburg gave an overview of CNS antibodies and associated synaptic pathophysiologies including stiff person syndrome (SPS), neuromyelitis optica (NMO) and limbic encephalitis. He introduced an animal model of SPS achieved by passive transfer of GAD-65 antibodies in rodents. Furthermore, he developed a model system for Batten disease, a neuropsychiatric disease of school-aged children using *Cln3^-/- ^*mice. In a third model, NMO pathophysiology was induced by intrathecal injection of NMO IgG or anti-AQP-4 antibodies together with complement.

The outbreak of hemolytic uremic syndrome (HUS) and diarrhea caused by Shiga toxin-producing *Escherichia coli *O104:H4 (STEC) in Germany during May to July 2011 involved severe and characteristic neurologic manifestations. Kerstin Göbel from Münster (Sven Meuth's lab) presented clinical and paraclinical data of 7 female, adult patients and single-cell patch-clamp recordings from murine thalamic relay neurons demonstrating a direct cytotoxic effect of Shiga toxin 2 (Stx 2) on thalamic but not cortical neurons. This primary involvement of the thalamus in STEC infection might be related to the gender-specific high expression of the Stx 2 receptor globotriaosylceramide (GB3).

In further sessions, several presentations focused on degenerative diseases of the peripheral and central nervous system comprising studies on neuropathies, Parkinson's disease (PD), Huntington's disease (HD) and Alzheimer's disease (AD). In a comprehensive study on toxic neuropathies, Wolfgang Böhmerle from Berlin (lab of Matthias Endres) focused on the role of calcium in toxic neuropathies, either induced via taxols and endoplasmic reticulum stress or salinomycin which induced mitochondrial damage.

Gunnar Hargus from Münster analyzed the potential of human pluripotent stem cells in models of PD. Pluripotent stem cells can generate cells of the three germ layers, and have unlimited self-renewal capacity. Of particular interest are so called iPS cells which are generated by reprogramming of differentiating somatic cells via specific protocols.

Another study on PD was presented by Joachim Göbel from Günther Höglinger's group in Marburg (now Munich). The use of LUHMES cells to generate synuclein transfected tyrosin hydroxylase positive neurons as a model for cytotoxicity provides an elegant paradigm to test mechanism of disease or protective compounds.

Using GFP transgenic bone marrow chimera and APP PS1 transgenic mice, Karin Kierdorf from Marco Prinz's lab in Freiburg presented data on the distinct roles of microglia and myeloid cells in this model mimicking aspects of AD.

Gisa Ellrichmann from the group of Ralf Gold in Bochum gave a talk on the protective role of fumaric acid esters in the R6/2 and YAC128 models of HD.

Finally, Gwendolyn Behrend's presentation (from the lab of Magdalena Götz in Munich) focused on reactive glia dedifferentiation in neurodegeneration. She showed that local differentiation of astrocytes into neural stem cells is possible in acute lesions. In contrast, there is little to no de-differentiation in chronic models of AD.

### Contributions on stroke and vascular pathology

As in the past 2010 meeting, one key topic in the neurovascular field was again related to the role of immune mechanisms and inflammatory circuits in the pathophysiology of stroke. Peter Ludewig from Tim Magnus's group in Hamburg provided conclusive evidence of a beneficial function of CEACAM-1 during ischemic brain damage. CEACAM-1 knockout mice developed significantly larger infarctions after middle cerebral artery occlusion (MCAO) and this was due to enhanced blood-brain-barrier leakage and impaired neurovascular recovery.

By using different immunological mouse models Juliane Klehmet from the Charité in Berlin (group of Andreas Meisel) could show that the enhanced infiltration of CNS antigen specific T cells might be one important mediator of stroke-induced immunodepression. Interestingly, antibiotic treatment was able to reverse the priming of CNS antigen-specific T cells in this setting and thus, may have the potency to counteract harmful immunodepression.

Christiane Albert-Weißenberger from Christoph Kleinschnitz's lab in Würzburg presented preliminary data on the involvement of the proinflammatory kallikrein-kinin system after diffuse traumatic brain injury. Mice lacking the kinin receptor B1 but not kinin receptor B2 developed less severe neurological deficits when analyzed in the weight drop model. This protective effect, which was restricted to the later phase after brain trauma only, is probably related to reduced astroglia activation and the prevention of diffuse axonal damage.

Finally, Martin Busch, doctoral student in the group of Alma Zernecke at the Rudolf-Virchow Center in Würzburg, explained how miRNA could be involved in the regulation of dendritic cell function during atherosclerosis.

A classical neuroprotection approach was presented by Christoph Harms, Junior Professor at the Charité in Berlin, who talked about an interesting molecule called apoptosis repressor with card domain (ARC) and its pleiotropic functions in in vitro and in vivo models of ischemic stroke.

Another focus of this year's meeting was on intracerebral hemorrhage. In accordance with the findings in brain ischemia, Bernd Kallmünzer from Erlangen showed that the hematopoietic growth factor G-CSF is also able to ameliorate functional deficits in models of intracranial bleeding (ICB). The underlying mechanisms, amongst others modulation of inflammation or proliferation of stem cells, are currently under investigation.

Arne Lauer from Christian Foerch's group in Frankfurt discussed the clinically relevant question whether or not the novel anticoagulant Dabigatran fosters hematoma growth after experimentally induced ICB in rodents. Interestingly, Dabigatran did not lead to increased bleeding volumes neither in the collagenase model nor after laser-induced rupture of cerebral blood vessels.

Two additional studies addressed the hot topic of vascular dementia. Anna Karpinska reported on the latest findings from the group of Martin Dichgans in Munich. By establishing a novel fluorescence-based single molecule bioassay (SIFT) they are now able to characterize NOTCH aggregation processes in vitro which could lead to a better understanding of the rare CADASIL syndrome.

Stefanie Schreiber from the German Center for Neurodegenerative Disorders (DZNE) in Magdeburg made use of spontaneously hypertensive stroke-prone rats (SHRSP) to study the pathologic cascade of cerebral microangiopathy (small vessel disease). She could show that intravascular accumulations of erythrocytes, parsimoniously interpreted as stases and associated with blood-brain barrier disturbances, is the initial step of this pathological cascade which is subsequently followed by microbleeds, mircothromboses and (hemorrhagic) tissue infarctions.

Finally, Maria Adele Rüger from Cologne reported on an elegant PET-based imaging method to non-invasively trace the mobilization of endogenous neural stem cells after focal brain ischemia.

## Competing interests

The authors declare that they have no competing interests.

## Authors' contributions

CK, SGM, TM, TK, and RL wrote the paper. All authors read and approved the final manuscript.

